# Chemical composition of *Nigella sativa* Linn: Part 2 Recent advances

**DOI:** 10.1007/s10787-016-0262-7

**Published:** 2016-04-11

**Authors:** M. Akram Khan, M. Afzal

**Affiliations:** Biomolecular Science Centre, Sheffield Hallam University, Howard Street, Sheffield, S1 1WB UK; Department of Biological Sciences, Faculty of Science, Kuwait University, Kuwait City, Kuwait

**Keywords:** *Nigella sativa* Linn, Chemical composition, Alkaloids, Organic synthesis, Medicinal properties

## Abstract

The black cumin or *Nigella sativa* L. seeds have many acclaimed medicinal properties such as bronchodilatory, hypotensive, antibacterial, antifungal, analgesic, anti-inflammatory and immunopotentiating. This review article is an update on the previous article published on *Nigella sativa* L. in this journal in 1999. It covers the medicinal properties and chemical syntheses of the alkaloids isolated from the seeds of the herb.

## Introduction

**Fig. 1:**
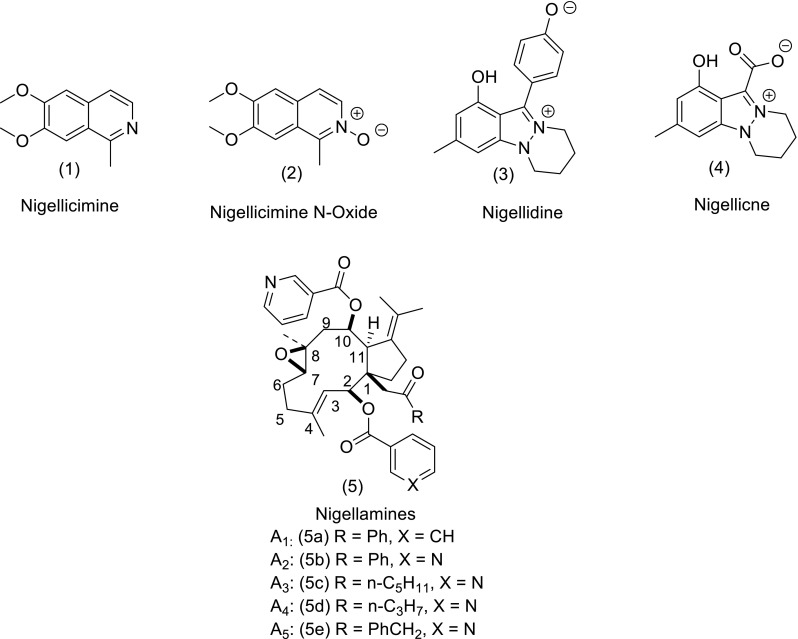
Structures of alkaloids isolated from *Nigella sativa* L.

**Fig. 2:**
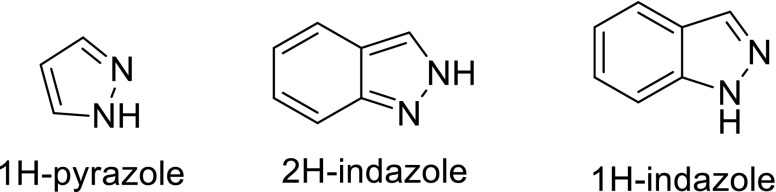
Types of indazole ring compounds

 The chemical composition and biological properties of *Nigella sativa* L. have previously been reviewed (Khan 1999; Paarakh 2010; Ahmed and El-Mottaleb 2013). In the previous review (Khan 1999) were reported the large variety of organic compounds that are present in the seeds of *N. sativa* L. The seeds of this herb are used in the Middle East and South Asian countries for the treatment of a large variety of ailments and are accepted as a panacea. For example, the seeds or oil from the seeds have been used to control diabetes, hypertension, cancer (leukeamia, liver, lung, kidney, prostate, breast, cervix, skin), inflammation, hepatic disorder, arthritis, kidney disorder, cardiovascular complications and dermatological conditions (Khan et al. 2003b, 2011). A GC–MS analysis of the seed extract has shown it to be a mixture of eight fatty acids and 32 volatile terpenes. The major terpenes, thymoquinone (TQ), dithymoquinone (DTQ), trans-anethol, p-cymene, limonine, and carvone have been identified (Nickavar et al. 2003). TQ and DTQ are both cytotoxic for various types of tumors (Worthen et al. 1998). In addition diterpenes, triterpene and terpene alkaloids have been identified in *N. sativa* seeds. The methanolic extract of the seeds contain two types of alkaloids whilst the major principal active ingredient isolated from the volatile oil of *N.sativa* L. is TQ. Since *N.sativa* L. acts as a panacea exhibiting a wide variety of pharmacological actions discussed previously and updated in this report, interest has arisen in the total synthesis of the alkaloids isolated having the isoquinoline and indazole motifs. The isoquinoline alkaloids include nigellicimine (1) and nigellicimine-N-oxide (2), and the indazole alkaloids include nigellidine (3) and nigellicine (4) (Fig. 1). Since the previous review several new dolabellane-type diterpene alkaloids, nigellamines A_1_–A_5_ (5) have also been isolated from the methanolic extract of the seeds of *N. sativa* L. which have also received synthetic interest (Fig. 1). In this update on *N. sativa* we want to discuss the chemistry of these various alkaloids and TQ under separate headings (Fig. 2).

### Pyrazole and indazole ring systems

**Fig. 3:**
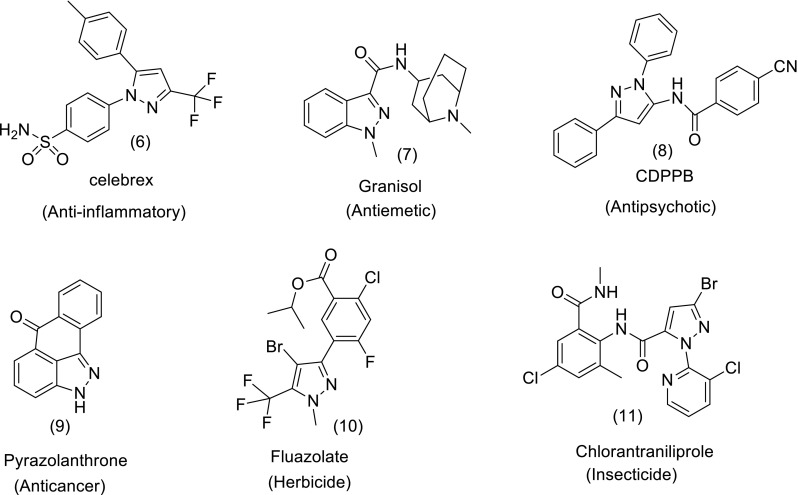
Structures of some pharmaceuticals and agrochemicals with indazole and pyrazole motifs

Indazole and pyrazole motifs are embedded in numerous pharmaceuticals and agrochemicals with a broad range of biological activities such as (6) (Penning et al. 1997), (7) (Plosker and Goa 1991), (8) (de Paulis et al. 2006), (9) (Okuno et al. 2004), (10) (Maxwell 2000) and (11) (Lahm et al. 2009) shown in Fig. 3.

As a result of these biological activities being associated with the presence of pyrazole and indazole pharmacore in therapeutic compounds, the two indazole alkaloids nigellidine (3) and nigellicine (4) have attracted the attention of synthetic organic chemists for their total syntheses. Thus multigram quantities of these two alkaloids can now be obtained via their total syntheses that should enable their individual therapeutic evaluation to be possible.

## Chemistry of the alkaloids and TQ in *Nigella sativa*

### Total synthesis of nigellidine (3)

**Scheme 1:**
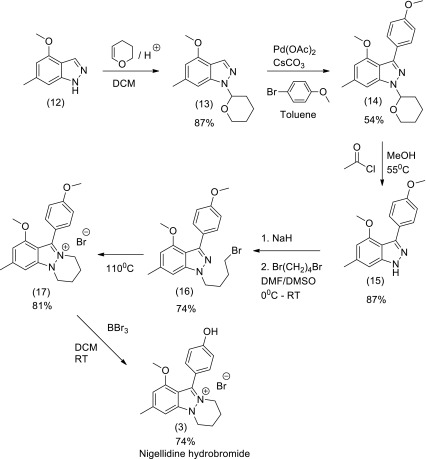
Total synthesis of nigellidine as the hydrobromide salt

The development of an efficient synthetic method using Pd(II)/Phen catalyst and conditions for the direct C-3 C–H arylation of (1H) indazole and pyrazole with ArI or ArBr was applied to the synthesis of nigellidine as shown in Scheme 1 (Ye et al. 2013). The THP derivative of the commercially available 4-methoxy-6-methyl-(1H)-indazole was reacted with 4-bromoanisole using the C-3 arylation reaction as a key step to form the adduct (14) in 54 % isolated yield on the gram scale. Deprotection of the tetrahydropyranyl (THP) group gave (15) which N-alkylation with 1,4-dibromobutane to afford (16) that underwent intramolecular cyclised to furnish the precursor (17). Demethylation of (17) by treatment with BBr_3_ afforded the natural product nigellidine (3) as the hydrobromide salt in an overall yield of 18 %.

### The total syntheses of nigellicine (4)

**Scheme 2:**
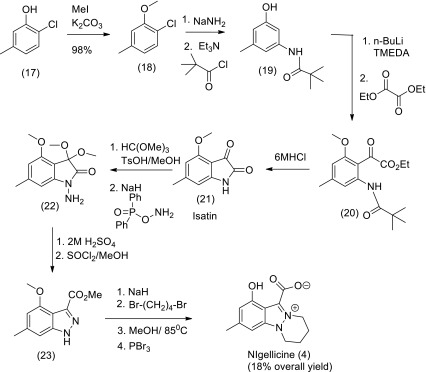
Total synthesis of nigellicine

To date there have been two total syntheses of nigellicine reported. In the first synthesis shown in Scheme 2 commercially available 2-chloro-5-methylphenol (17) was transformed into the protected amide (10) which on lithiation and acylation with diethyloxalate yielded the amide-ester (20) that cyclised on treatment with 6MHCl acid into the isatin (21) (Elliott et al. 2005). Protection of the keto group in isatin (21) as the dimethyl acetal (22) enabled direct amination at nitrogen to give the hydrazine derivative (22) which on treatment with aqueous acid rearranged into an indazole carboxylic acid that was esterified via an acid chloride into (23). Alkylation of indazole ester (23) with 1,4-dibromobutane and subsequent intramolecular cyclisation produced a 4-methoxy derivative of nigellicine which was deprotected with PBr_3_ to give nigellicine in an overall yield of 18 %.

**Scheme 3:**
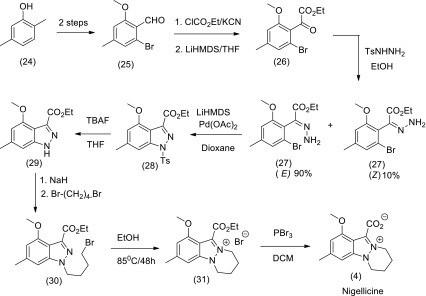
Second synthesis of nigellicine (4)

The second total synthesis of nigellicine (4) shown in Scheme 3 starts with commercially available 2,5-dimethylphenol (24) which was converted into 2-bromo-6-methoxy-4-methylbenzaldehyde (25) by Clive’s method (Inamoto et al. 2007). Treatment of aldehyde (25) with KCN and ethyl chloroformate in the presence of benzyltrimethylammonium chloride (BTAC) and 18-crown-6 in a mixture of water and 1,2-dichloroethane produced an intermediate cyanohydrin carbonate ester which was subsequently converted to a-ketoester (26) by LiHMDS-induced rearrangement. The reaction of (26) with p-toluenesulfonyl hydrazide gave the key intermediate hydrazone (27) as a mixture of *E*- and *Z*-isomers, which was separable by column chromatography to obtain the major trans isomer that was subsequently converted by Pd-catalysed cyclisation to the indazole (28). Alkylation of the deprotected compound (29) with 1,4-dibromobutane produced the intermediate (30) which underwent intramolecular cyclisation in hot ethanol to furnish the nigellicine ethyl ester hydrobromide salt (31). Finally treatment of ester (31) with PBr_3_ caused cleavage of the ester group and deprotection of the methoxy group to give nigellicine (4).

### Total synthesis of nigellamine A_2_ (5b)

The delabellane diterpenes are ubiquitous molecules that are produced by animals, plants, fungi and marine sources and have interesting array of biological activities. The alkaloids nigellamines A_1_–A_5_ which have been isolated from *N. sati*v*a* L. belong to the delabellane family of diterpenes and show potent lipid metabolism-promoting activity (Morikawa et al. 2004a). These biologically active alkaloids have complex structural features and have attracted the attention of synthetic organic chemists for their total synthesis.

**Scheme 4:**
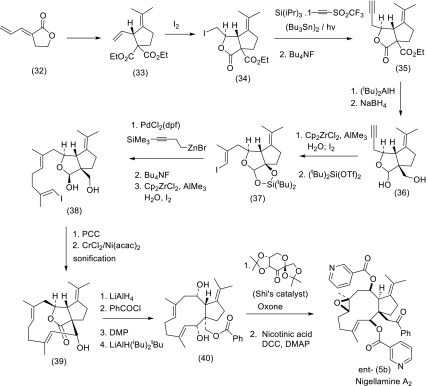
Total synthesis of nigellamine A_2_

One enantioselective total synthesis of nigellamine A_2_ has so far been reported (Bian et al. 2006). In this synthesis shown in Scheme 4 the starting lactone-diene (32) was transformed in three steps and on on multigram scale into the allylic ester (33) as a key intermediate. Iodolactonisation of diene (33) produced (34) which on radical alkynylation furnished the propynyl lactone (35). Desilylation and reduction of (35) yielded the propynyl lactol (36) which upon in situ iodination and subsequent silylation afforded the vinyl iodide (37) in good yield. The remaining carbon atoms of the nigellamine skeleton were constructed through cross coupling with alkyl zinc reagent and a repeat methylalumination-iodination sequence of reactions to afford substrate (38). Oxidation with pyridinium chlorochromate (PCC) gave an aldehyde at position C_2_ which upon sonification underwent Cr-mediated cyclisation with the vinyl iodide group at position

C_3_ to generate the 11-membered compound (39). Reductive opening of the lactone and selective acylation of the primary alcohol gave the substrate (40). Oxidation of (40) with Shi’s ketone catalyst and oxone proceeded region- and stereoselectively to produce the desired epoxide-diol as the major product which was acylated with nicotinic acid to furnish ent-nigellamine A_2_ (5b).

### Novel synthetic thymoquinone analogues

**Scheme 5:**
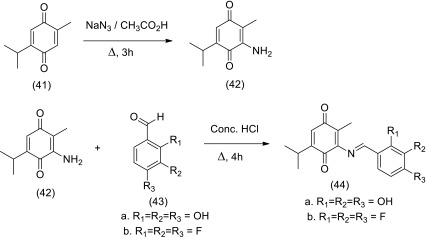
Synthesis of thymoquinone alanogues as anticancer agents

The compound 5-isopropyl-2-methyl-1,4-benzoquinone is known as thymoquinone (TQ) (41) shown in Scheme 5. TQ is the major active principle of the oil of *N. sativa* L. and has been shown to exhibit anti-tumor activity against breast, lung, prostrate, liver, colon and pancreatic cancer. Thus interest has arisen to synthesise more potent analogues of TQ. Recently reported are the novel analogues of TQ consisting of compounds (44a–b) were synthesised in two steps from TQ Sodium azide added to TQ in acetic acid to afford the reduced product (42) which on reaction with the aldehydes (43a–b) generated the Schiff bases (44a–b) (Yusufi et al. 2013). These analogues have shown superior proliferative activity, excellent chemo-sensitizing activity against pancreatic cancer in vitro and in combination with Gemcitabine.

One serious drawback with TQ is its toxicity at high doses and poor water solubility which limit its usage as a therapeutic agent. In order to alleviate this problem various types of nanocarrier for thymoquinone have been synthesised (Ravindran et al. 2010; Ganea et al. 2010; Alam et al. 2012; Singh et al. 2013). One recent study has reported the synthesis of PAG coated NIPAAM nanoparticles that are encapsulated with TQ for direct hepato-targeting. NIPAAM is a thermosensitive nanopolymer which is widely used as a successful drug delivery system against various diseases and PAG is a galactosylated moiety that targets the liver by interacting with asialoglycoprotein receptor (ASGP-R) present on the surface of hepatocytes and delivers the drug directly to the liver (Verma et al. 2013). The toxicity of the nanocarrier (NIPAAM) at this concentration is almost negligible and due to the size of the nanoparticle being smaller than the already reported nanothymoquinone.

This study clearly has demonstrated that the nanoparticles are able to carry bulk amounts of drug to the liver, and their direct targeting to ASGP-R receptors present on hepatocytes has resulted in significant hepatoprotection at a low dose level that is 1000 times lower than the naked TQ. This nanocarrier approach offers a promising prospect for the future against various liver diseases.

## Biological activities of *Nigella sativa*

### The anti-inflammatory activities

**Fig. 4:**
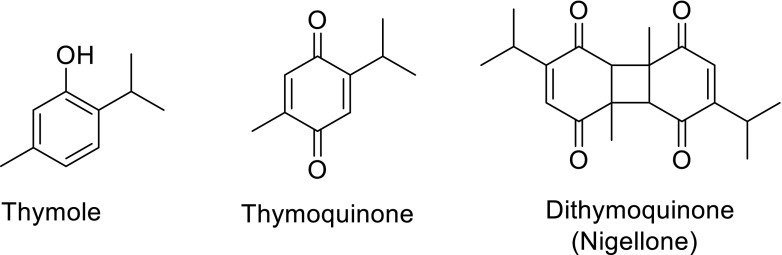
Chemical structures of principal active ingredient isolated from the volatile oil of *Nigella* *sativa* L.

In animal studies *N. sativa* shows dose-dependent suppression of nociceptive pain response and cestocidal activity. These activities are shown by TQ that acts through indirect activation of the supraspinal mu(1)- and kappa-opioid receptor subtypes (Abdel-Fattah et al. 2000; Akhtar and Riffat 1991). The antihypertensive principal TQ and other constituents of *N. sativa* are also protective agents against the chromosomal aberrations induced by schistosomiasis (Aboul-El-Ela 2002; El Tahir et al. 1993a). These compounds are used in the control of arterial blood pressure, anticholinergic, antihistaminic, tracheal relaxation, control of asthma and in the treatment of other allergic diseases (Ahmed and El-Mottaleb 2013; Al-Majed et al. 2001; Boskabady et al. 2004; Kalus et al. 2003; Steinmann et al. 1997) (Fig. 4).

Nigellone (dithymoquinone) is the carbonyl dimer of TQ present in *N. sativa* and it inhibits the release of histamine giving relief in asthmatic conditions (Chakravarty 1993; El Tahir et al. 1993b). The spasmolytic and bronchodilator activities of *N. sativa* are mediated possibly through calcium channel blockade (Gilani et al. 2001). Physologically important activities shown by *N. sativa* include analgesic, anti-inflammatory, antimicrobial, antifungal and antibacterial effects (Hanafy and Hatem 1991; Khan et al. 2003a; Morsi 2000) and CNS activity of its aqueous extract and volatile oil components (Al-Ghamdi 2001; Al-Naggar et al. 2003; Hajhashemi et al. 2004; Haq et al. 1995). The neuroprotective activity of *N. sativa* on neurotransmitter leading to antiepileptic activity has also been described (Arafa et al. 2013). TQ, through an opioid receptor-mediated, increases in GABAergic tone, exhibits anticonvulsant activity in the petit mal epilepsy (Hosseinzadeh and Parvardeh 2004).

### Antiulcer and anticancer properties

Ethanol induced ulcer in rats has been reduced by *N. sativa* extracts (El-Dakhakhny et al. 2000a, b). Ischaemia/reperfusion are linked by free radical generation and this could be controlled by an administration of TQ which could offer gastroprotective effects against gastric lesions (El-Abhar et al. 2003). The chemosensitising effect of TQ in the treatment of 5-Fluorouracil induced gastric cancer has been reported (Lei et al. 2012).

### Hepato-protective antioxidant activities

The aqueous extract of *N. sativa* (NS) is hepato-protective against carbon tetrachloride induced oxidative hepatic damage suggesting powerful antioxidative properties of NS extract (Al-Ghamdi 2003; El-Dakhakhny et al. 2000a, b; Mansour et al. 2001; Meral and Kanter 2003). NS protects liver by inhibiting enzyme leakage from hepatocytes caused by toxic substances such as carbon tetrachloride (Kanter et al. 2003a). Through its antioxidant action, TQ is known to inhibit 5-lipoxigenase and 5-hydroxy-eicosatetraenoic acid (5-HETE) products suggesting its use in inflammatory pathogenesis (El-Dakhakhny et al. 2002a). Hyperhomocysteinemia (HHcy) has been linked with oxidative stress. Therefore, NS has been demonstrated to improve total antioxidant status in rats treated with methionine induced HHcy (El-Saleh et al. 2004). The oxygen free radical generated by gentamicin pathogenesis causing hepatotoxicity and nephrotoxicity are quenched by oil and seeds of NS (Ali 2004) and the ethanolic extracts of NS have the potential to protect against gama-radiation induced oxidative damage (Rastogi et al. 2010). It has been reported that TQ inhibits the leakage of hepatic enzymes and the intracellular depletion of GSH protecting liver (Daba and Abdel-Rahman 1998).

### Immunomodulatory effect

NS has established immunosuppressive and cytotoxic properties (Islam et al. 2004) and the pharmacological and therapeutical properties of NS have been reviewed by many workers (Ahmad et al. 2013; Ali and Blunden 2003; Swamy and Tan 2000). The splenocyte proliferation, macrophage function, and NK anti-tumor activity of NS have revealed the potent immunomodulatory properties of the Nigella seeds (Majdalawieh et al. 2010). The NS seed oil also shows hepatoprotective action against hypervitaminosis A and humoral immune responses and non-specific cellular immune responses (Al-Suhaimi 2012; Al-shatwi 2014). The immune modulating effect of NS is mediated through direct stimulation of macrophage phagocytic activity or lymphocytes activation (Fararh et al. 2004; Haq et al. 1999).

NS is a known immune stimulant that protects against many pathological conditions (Corder et al. 2003; Fararh et al. 2004). Thus, the powerful antioxidative and protective properties of TQ in proteinuria and hyperlipidemia associated with nephrotic syndrome have been evaluated (Badary et al. 2000). Along with TQ and other terpenoid compounds such as carvacrol, trans-anethole and 4-terpineol with antioxidant properties have been reported for NS (Burits and Bucar 2000). Carvacrol is a known inhibitor of human neutrophil elastase and may be useful agent in phytotherapy for injuries such as chronic obstructive pulmonary disease and emphysema (Kacem and Meraihi 2006). The volatile NS oil shows anti-oxytocic and sperm characteristics due to its antioxidant activities (Mansour et al. 2013; Aqel and Shaheen 1996).

### Effect on blood sugar and lipid profile

Streptozotocin (STZ) treated animals respond to NS extracts with normalizing blood glucose through extrapancreatic actions rather than by stimulated insulin release and ascertain to be protective against type-2 diabetes (El-Dakhakhny et al. 2002b; Fararh et al. 2002, 2004; Hawsawi et al. 2001). The significant increase in lipid peroxidation by STZ is also controlled by NS and has protective effect in diabetes by decreasing oxidative stress and regeneration/proliferation of the beta-cells in the islets of Langerhans (Kanter et al. 2003b, 2004). A petroleum ether extract of NS exhibits insulin-sensitizing activity (Le et al. 2004) and the mechanism of NS extract in the control of diabetes has ben shown to be through controlled insulin release (Rchid et al. 2004). At the same time, amendment in the blood lipids profile has been suggested by the use of NS extracts (El-Dakhakhny et al. 2000a, b). Arachidonic acid induced blood platelet aggregation and blood coagulation are inhibited by NS indicating its potential use in thrombosis (Enomoto et al. 2001). TQ is involved in the inhibition of arachidonic acid generated eicosanoids and lipid peroxidation (Houghton et al. 1995).

### Effect on arthritis

In human, TQ has been shown to be effective in rheumatoid arthritis (Gheita and Kenawy 2012). Inhibition of arachidonic acid generated eicosanoids (thromboxane B2, leukotriene B4) supports the use of NS for the treatment of rheumatoid arthritis and other inflammatory diseases (Houghton et al. 1995). TQ has been implicated in bone healing in an animal model (Kirui et al. 2004). Inhibition of leukotrienes through 5-lipoxygenase and LTC4 synthase activities in eicosanoid pathway has been well documented (Mansour and Tornhamre 2004).

### Anticancer activity of TQ

**Fig. 5:**
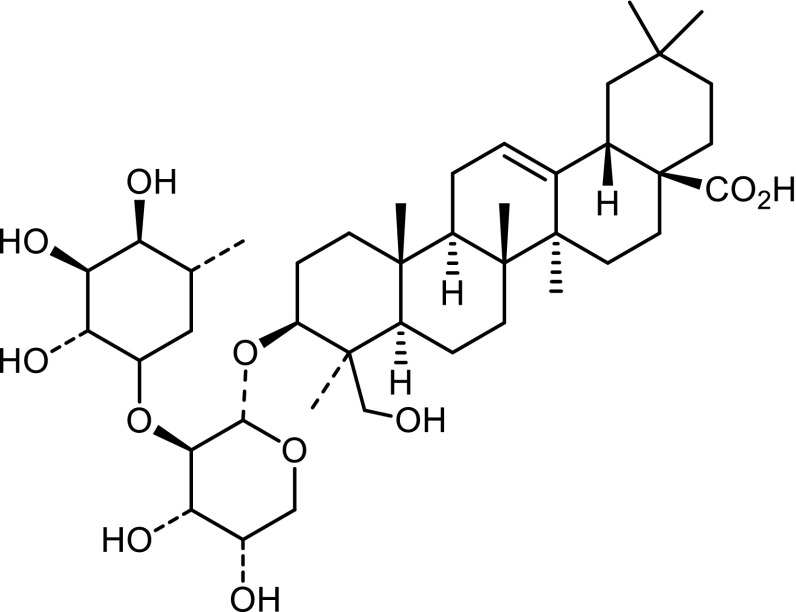
Chemical structure of α-hederin

A number of antitumor compounds have been identified from NS. These compounds are TQ, alpha-hederin a triterpene, isopropylmethylphenols and dollabelane-type diterpene alkaloid nigellamine A3, A4, A5 and C (Kumara and Huat 2001; Michelitsch et al. 2004; Morikawa et al. 2004a, b). Thus, numerous types of cancers such as Ehrlich ascites carcinoma (EAC), Dalton’s lymphonia ascites (DLA) and Sarcoma-180 (S-180) cells, colon carcinoma, pancreatic carcinoma and hepatic carcinoma have been treated with NS extracts in vitro (Salomi et al. 1992; Samarakoon et al. 2010). Changes in intracellular GSH and redox status for mitochondrial function are important factors in the mechanism of alpha-hederin induced cell death (Swamy and Huat 2003) (Fig. 5).

The NS extract exerts anti-hepatocarcinoma effect through modulation of apoptosis (Samarakoon et al. 2012). The regulation of pro- and anti apoptic genes by NS has been demonstrated in treating cervical cancer (Shafi et al. 2009). In many cases the antitumor activity of NS seeds has been attributed to the volatile component thymoquinone (structurally related to tert-butylhydroquinone, a potent antioxidant) that has the potential to protect rat liver against diethynitrosamine (DEN) induced hepatocarcinogenesis (Iddamaldeniya et al. 2003). It also improves the therapeutic efficacy of ifosfamide by decreasing nephrotoxicity and improving antitumor activity (Badary 1999; Saleem et al. 2012).

TQ also affects the benzo-a-pyrene induced clastogenic activity in rats and 20-methylcycloanthrene induced fibrocarcinoma is inhibited by TQ present in NS extracts (Badary et al. 2007; Badary and Gamal El-Din 2001). While supplementation by NS and honey in the treatment of methylnitrosourea induced inflammation, carcinogenesis and oxidative stress has been reported (Mabrouk et al. 2002), the lipid peroxidation induced liver damage in diabetic rats has also been mentioned (Meral et al. 2001).

The pro-oxidant nitric oxide production is inhibited by NS extracts validating the fact that NS has anti-inflammatory activities (Mahmood et al. 2003). Model in vivo experiments with Schistosomiasis mansoni infected mice have concluded that NS extract have a great protective potential against oxidative stress protecting liver (Mahmoud et al. 2002). The mode of action of TQ against cancer has been suggested to be through its antioxidative properties and interaction with DNA synthesis. The antioxidant and pro-oxidant properties of TQ have been substantiated by augmented TQ mediated scavenging of superoxide anion (Badary et al. 2003). However, presence of the phenolic compounds in NS, such as vanillic acid, could also contribute to the antioxidant properties of NS. These compounds may also be responsible for its antimutagenic activities (Bourgou et al. 2008; Khader et al. 2010). TQ exhibits advanced antimyeloma activity in MDN and XD2 multiple myeloma malignant plasma cells (Badr et al. 2011). However, the mechanism of chemotaxis of malignant plasma cells is not well defined.

### Effect of TQ on pancreatic carcinoma (PC)

PC is one of the most deadly cancers with almost invariably fatal consequences. TQ has antitumor activity against PC. To combat PC, the dose of TQ has to be high. Therefore, many attempts have been made to study structure activity relationships by synthesizing TQ analogs and some of these compounds have potent antitumor activity against PC (Banerjee et al. 2010). Gemcitabine- or oxaliplatin-induced activation of NF-kappaB is inhibited by TQ, resulting in the chemosensitization of pancreatic tumors to conventional therapeutics (Banerjee et al. 2009). Progressive apoptosis is also inhibited by NS (Corder et al. 2003).

### Co-administration of NS with other substances

Cisplatin is a widely used drug that induces kidney toxicity. It has been established that when cisplatin is co-administered with NS, the nephrotoxicity is reduced (El-Daly 1998; Nair et al. 1991; Ulu et al. 2012). A co-administration of NS with green tea extract prevents cytotoxicity of organophosphorus compounds (Korany and Ezzat 2011). Co-administration of saffron with NS in the treatment of chemical carcinogenesis has also been reported (Salomi et al. 1991).

## Breast cancer

It is one of the most common causes of death in women and there is no effective treatment except mastectomy. Many substances have been shown to have mammary anticancer activity and among these are melotonin and retinoic acid. NS has been examined in animals exposed to 7,12-di-methylbenz(a)anthracene (DMBA), mammary cancer causing substance which showed NS reduces the carcinogenic effects of DMBA (El-Aziz et al. 2005). Inactivation of MCF-7 breast cancer cells has been demonstrated by NS extracts (Farah and Begum 2003).

### Colon cancer

The molecular mechanism of action of TQ in colon cancer has been suggested. Thus, colon cancer is inhibited in G1 phase cell cycle and apoptosis is mediated by TQ (Gali-Muhtasib et al. 2004). The 1,2-dimethylhydrazine (DMH), colon cancer inducer, damage erythrocytes has been reported and NS has the ability to detoxify DMH (Harzallah et al. 2012; Worthen et al. 1998). The preneoplastic lesions for colon cancer have been investigated and found that colon cancer in post-initiation stage can be prevented by volatile components of Nigella seeds (Salim and Fukushima 2003).

## Conclusion

The Islamic claim made by prophet Muhammad over 1400 years ago that “black seed (*N. sativ*a Linn) has the cure for all deseases” has a much more meaningful and acceptable dimension to it given the overwhelming scientific data obtained, as outlined in the reviews, that supports it. The alkaloids present in *N. sativa* Linn could now be obtained by total synthesis and the study of their pharmacological properties should make very interesting research studies for the future.
